# IVIG activates FcγRIIB-SHIP1-PIP3 Pathway to stabilize mast cells and suppress inflammation after ICH in mice

**DOI:** 10.1038/s41598-017-15455-w

**Published:** 2017-11-14

**Authors:** Gokce Yilmaz Akyol, Anatol Manaenko, Onat Akyol, Ihsan Solaroglu, Wing Mann Ho, Yan Ding, Jerry Flores, John H. Zhang, Jiping Tang

**Affiliations:** 1 Departments of Basic Science, Loma Linda University, Loma Linda, CA USA; 2 Departments of Neurology, University of Erlangen-Nuremberg, Erlangen, Germany; 3 Department of Anesthesiology, Loma Linda University, Loma Linda, CA USA

## Abstract

Following intracerebral hemorrhage (ICH), the activation of mast cell contributes to brain inflammation and brain injury. The mast cell activation is negatively regulated by an inhibitory IgG-receptor. It’s signals are mediated by SHIP (Src homology 2-containing inositol 5′ phosphatase), in particular SHIP1, which activation leads to hydrolyzation of PIP3 (Phosphatidylinositol (3,4,5)-trisphosphate (PtdIns(3,4,5)P_3_, leading to the inhibition of calcium mobilization and to the attenuation of mast cell activation. Intravenous immunoglobulin (IVIG) is a FDA-approved drug containing IgG. We hypothesized that IVIG will attenuate the ICH-induced mast cell activation via FcγRIIB/SHIP1 pathway, resulting in a decrease of brain inflammation, protection of the blood-brain-barrier, and improvement of neurological functions after ICH. To prove this hypothesis we employed the ICH collagenase mouse model. We demonstrated that while ICH induced mast cell activation/degranulation, IVIG attenuated post-ICH mast cell activation. Mast cell deactivation resulted in reduced inflammation, consequently attenuating brain edema and improving of neurological functions after ICH. Furthermore using siRNA-induced *in vivo* knockdown approach we demonstrated that beneficial effects of IVIG were mediated, at least partly, via SHIP1/PIP3 pathway. We conclude that IVIG treatment represents a promising therapeutic approach potentially able to decrease mortality and morbidity after ICH in experimental models.

## Introduction

Spontaneous intracerebral hemorrhage (ICH) is a subtype of stroke, accounting for 15 to 20% of all stroke types. While the high mortality (>40%) and morbidity (>75%) makes ICH a challenging problem, there are no effective therapies for ICH patients^1–3^.

Mast cells are located along blood vessels in the brain^4^. Mast cell activation triggers various pathological processes. While the activation of mast cells after stroke is well established, the events leading to the activation have been only poorly investigated^5–7^. Assumable the rapid increase of IgE level, induced by the blood entry in the brain parenchyma^7^, the release of damage-associated molecular patterns (DAMPs) induced by physical injury and/or sheer stress induced by growing hematoma contribute to the rapid activation of mast cells after ICH^8–10^. After stroke the activation of mast cells results in inflammation leading to blood–brain barrier disruption, brain edema, and hemotoma expansions^5,6,11,12^. Mast cells activation is regulated by several activating receptors and one inhibitory IgG receptor, FcγRIIB^13,14^. The receptor contains intracytosolic immunoreceptor tyrosine-based inhibition motifs (ITIM) which are important for down-modulating immune responses^15^. Activation of ITIM containing receptors recruits Src homology 2 domain-containing inositol 5- phosphatase 1 (SHIP1) which dephosphorylates phosphatidylinositol 3,4,5 trisphosphate and terminates PI3K-mediated signaling pathways, diminishing the mast cell activation (Supplemental Fig. 1)^16^.

IVIG is an FDA-approved immunotherapeutic blood product that is formed from a pooled plasma of healthy donors and contains mainly IgG^17^. After ischemic stroke or traumatic brain injury, IVIG treatment improved BBB integrity, decreased cerebral infarct areas and brain edema as well as attenuated production of pro inflammatory cytokines^18,19^. The crucial mechanism, underlying IVIG induced protection, is an activation of FcγRIIB receptor, which decreases inflammatory cytokines production^20^. The anti-inflammatory effects of IVIG treatment were not observed in FcγRIIB-deficient mice^21^.

These observations led us to the hypothesis that IVIG may activate FcγRIIB receptor and attenuate mast cell activation in mice after ICH. We also hypothesized that IVIG induced mast cell deactivation may diminish post ICH inflammation and BBB disruption, consequently improving neurological functions. We suggested that beneficial effects of FcγRIIB receptor activation may be meditated by SHIP1-PIP3 pathway (for details see Supplemental Material).

## Results

### Mortality

The mortality rate in untreated animals is 10.6%. No statistical difference was found between experimental groups (Table 1 in Supplemental Material).

### Intraperitoneal administration results in increased levels of IVIG in the blood of mice

Intraperitoneal administration of IVIG resulted in significant increase of IVIG in the blood of mice, as evaluated by ELISA 24 hours after the drug administration. The effect was dose-dependent. A higher level of IVIG was detected in the blood of mice treated with high dose compared to the animals treated with low dose of IVIG (Supplemental Fig. 2).

### IVIG attenuated brain edema and BBB dysfunction without affecting on hematoma volume

The effects of treatment on hematoma volume was evaluated at 24 and 72 hours after ICH. IVIG treatment did not change the hematoma volume in this study (Supplemental Fig. 3).

**Figure 1:**
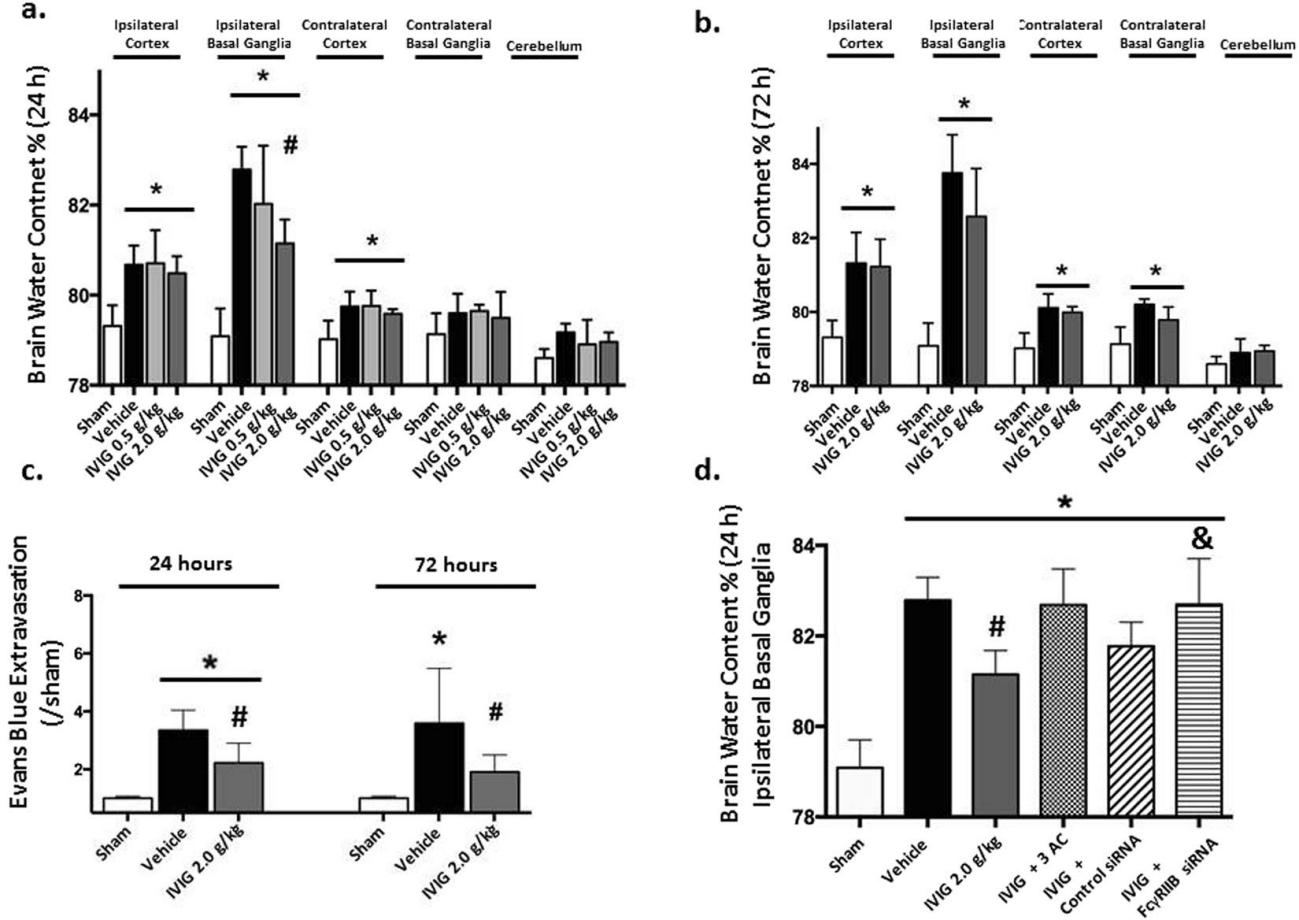
IVIG attenuated BBB disruption after ICH without affecting the hematoma volume. ICH increased water content in brain of ICH- compared to sham-operated animals evaluated at 24 (**a**) and 72 hours (**b**) after ICH. IVIG significantly attenuated the ICH-induced increase of brain water content in ipsilateral basal BBB at 24 (**a**) and shown the strong tendency to improvement at 72 hours (**b**) after ICH. Additionally the treatment attenuated post-ICH extravasation of Evans Blue Stain in the ipsilateral hemisphere at 24 and 72 hours after ICH (**c**). Knockdown of the FcγRIIB receptor or inhibition of the SHIP1 via 3AC (a SHIP1 inhibitor, 3α-aminocholestane), reversed effects of IVIG treatment on brain edema in ipsilateral basal ganglia evaluated at 24 hours after ICH. Scramble RNA (control siRNA) used as negative control did not show any effect on brain water content (**d**). (**a**) Brain Water Content at 24 hours (sham n = 6, vehicle n = 7, IVIG (0.5 g/kg) n = 6, IVIG (2 g/kg) n = 6) (**b**) 72 hours. (sham n = 6, vehicle n = 6, IVIG (2 g/kg) n = 6) (**c**) Evans Blue extravasation at 24 and 72 hours. (**d**) Effects of FcγRIIB or SHIP1 inhibition on the IVIG-induced attenuation of brain edema (sham n = 6, vehicle n = 7, IVIG (0.5 g/kg) n = 6, IVIG (2 g/kg) n = 6, IVIG + 3AC n = 6, IVIG + control siRNA n = 6, IVIg + FcγRIIBsiRNA n = 6). Values are expressed as mean ± SD. *significant vs. sham, ^#^significant vs. vehicle, ^&^significant vs. IVIG (2 g/kg), p < 0.05 ANOVA, Tukey Test.

Collagenase-induced ICH caused significant elevation of water content in the brains of ICH animals compared to sham operated animals both at 24 and 72 hours after ICH induction (Fig. 1a,b). Both low (0.5 g/kg) and high (2 g/kg) doses of IVIG reduced the ICH-induced increase of brain water content in the ipsilateral basal ganglia at 24 hours after ICH, however the significance was only reached in the high dose group (P < 0.05, compared with vehicle, Fig. 1a).

Furthermore the strong tendency to the reduction of brain water content by high dose IVIG was observed at 72 hours after ICH. The tendency did not reach statistical significance (Fig. 1b).

Additionally the effect of IVIG treatment on BBB integrity was evaluated by Evans Blue assay. Significant accumulation of Evans Blue stain was observed in ipsilateral hemisphere 24 and 72 hours after ICH (Fig. 1c). The treatment with high dose of IVIG reduced accumulation of Evans Blue significantly in ICH animals (Fig. 1c).

Effect of FcγRIIB-SHIP1 pathway manipulation on ICH induced brain edema was seen most prominently in ipsilateral basal ganglia (Fig. 1a). Inhibition of FcγRIIB in combination with IVIG administration reversed effects of IVIG on brain edema at 24 hours after ICH (P > 0.05, compared with vehicle, Fig. 1d). SHIP1 inhibition show the strong tendency to aggravation of brain edema (Fig. 1d). Neither IVIG nor inhibition of the FcγRIIB-SHIP1 pathway induced statistical significant changes in another brain compartments (Fig. 1a).

### IVIG improved neurological functions after experimental ICH

**Figure 2:**
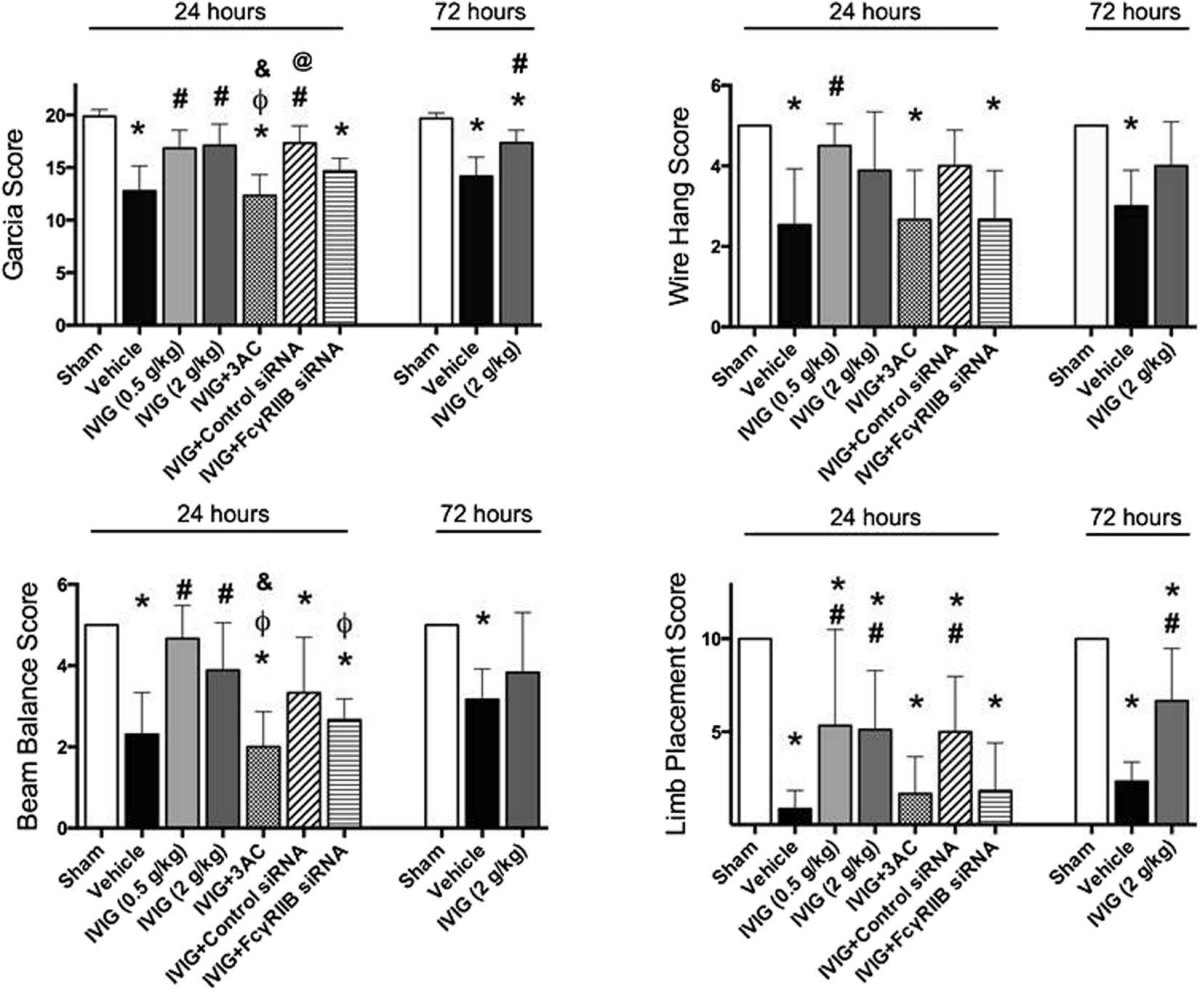
Effects of IVIG treatment on post-ICH neurological functions. ICH induced significant neurological dysfunction evaluated 24 and 72 hours after ICH by (**a**) Modified Garcia Score (**b**) Wire Hang (**c**) and Beam Balance Tests **d**) Limb Placement Test. IVIG improved neurological functions of ICH animals both 24 and 72 hours after ICH. FcγRIIB or SHIP1 inhibition reversed effects of IVIG treatment 24 hours after ICH. (sham n = 8, vehicle n = 13, IVIG (0.5 g/kg) n = 6, IVIG (2 g/kg) n = 9, IVIG + 3AC n = 9, IVIG + control siRNA n = 6, IVIg + FcγRIIBsiRNA n = 6) and at 72 hours (sham 6, vehicle n = 6, IVIG (2 g/kg) n = 6). Values are expressed as mean ± SD. *significant vs. sham, ^#^significant vs. vehicle, ^ϕ^significant vs. IVIG (0.5 g/kg), ^&^significant vs. IVIG (2 g/kg), ^@^significant vs. 3AC, p < 0.05 ANOVA, Tukey Test.

Compared with sham-operated, all ICH animals showed significant neurological deficits (Fig. 2a–d). Both low (0.5 g/kg) and high (2 g/kg) doses of IVIG improved ICH-impaired neurological functions evaluated 24 hours after ICH. Furthermore, 72 hour after ICH high dose of IVIG (2 g/kg) improved neurological functions evaluated by modified Garcia and Limb Placement tests (Fig. 2a,d). The inhibition of SHIP1 via 3AC (SHIP1 inhibitor, 3α-aminocholestane) or *in-vivo* knockdown of FcγRIIB via siRNA reversed the beneficial effects of IVIG (Fig. 2). Scramble RNA used as negative control (Control RNA) did not affect IVIG improved neurological functions.

### ICH induced time-dependent degranulation of mast cells

**Figure 3:**
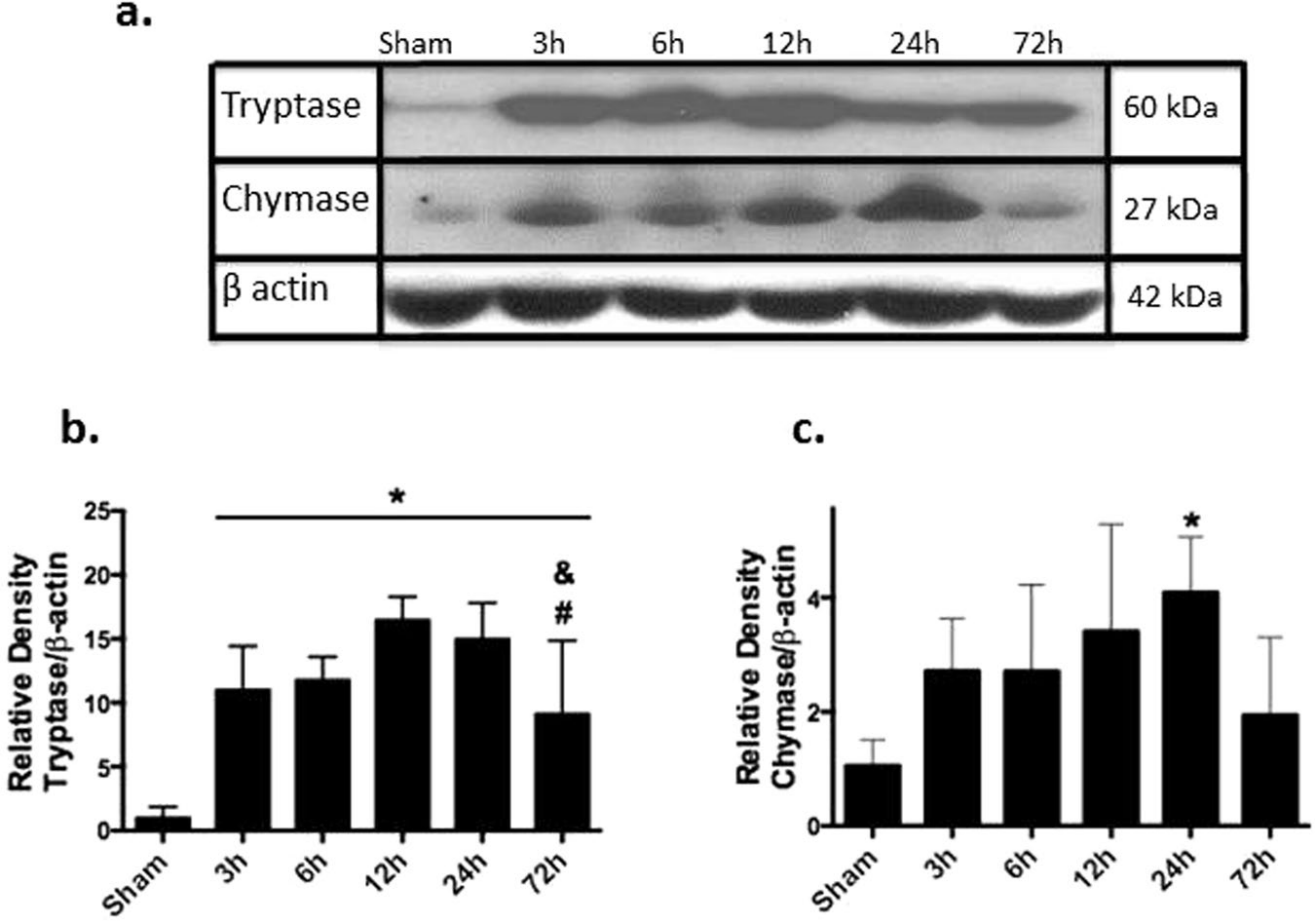
ICH increased time dependently expression of mast cell mediator tryptase and chymase. (**a**) Representative western blot. The regions of interests on the membranes were separated and proceeded as described in “Material and Methods” section. Expression of tryptase (**b**) and chymase (**c**) in ICH animals at 3, 6, 12, 24, 72 hours normalized to sham operated animals (tryptase n = 6, chymase n = 5) Values are expressed as mean ± SD. *significant vs. sham, ^#^significant vs. 12 hours, ^&^significant vs. 24 hours, p < 0.05 ANOVA, Tukey Test.

As evaluated by western blot study, ICH induced time-dependent release of mast cell mediators as a sign of mast cell activation and degranulation (Fig. 3a, a representative western blot). Compared to sham operated animals, an increase of the tryptase production was observed as early as 3 hours after ICH. The production remained upregulated until 72 hours after ICH (Fig. 3b). A tendency to the increase of the chymase production was observed 3 hours after ICH. The increase reached statistical significance 24 hours after (Fig. 3c).

### IVIG treatment prevented mast cell degranulation

**Figure 4:**
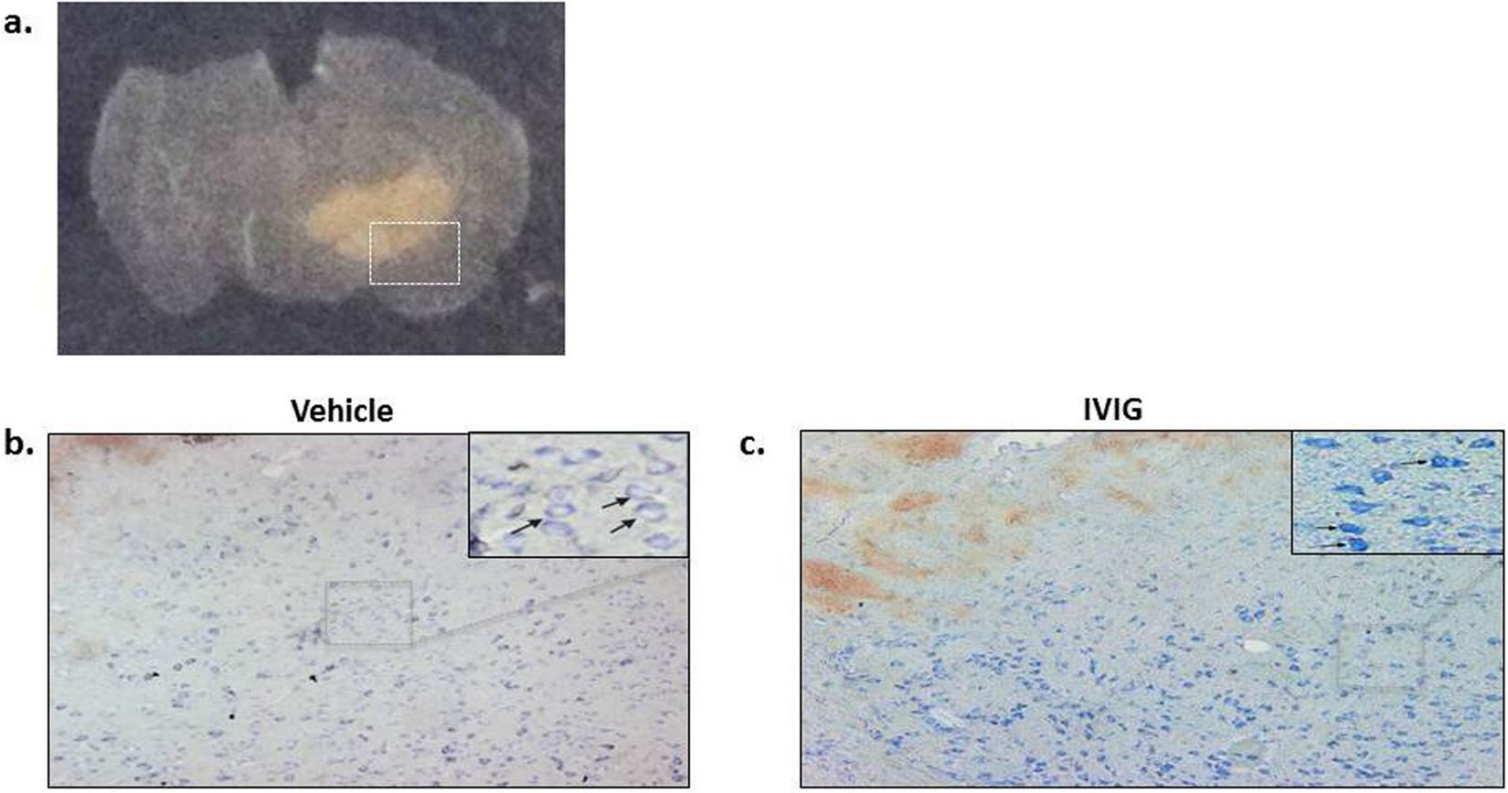
Effects of IVIG treatment on ICH induced mast cell degranulation and activation. (**a**) Coronal brain section showing the region on mast cell visualization. Toluidine Blue staining of mast cell in the brain of sham operated (**b**) ICH vehicle-treated (**c**) and ICH IVIG-treated animals (**d**) 24 hours after ICH. More mast cells were observed in brain of ICH compared to sham animals. While in the brain of IVIG treated animals granulated intensive stained cells were observed (**d**), most of the mast cells detected in brain of ICH animals (**c**) showed the clear signs of degranulation with less intensive Toluidine Blue staining and the appearance of ‘ghost’ cells.

Mast cell degranulation was investigated in the perihematomal region (Fig. 4a, blue quadrant represents the region of interest) by Toluidine Blue staining 24 hours after ICH. There were more Toluidine Blue positive cells in peri-hematomal region of ICH animals compared to the same brain region of sham-operated animals (Fig. 4b,c respectively). While mast cells in the brain of untreated animals showed the clear signs of degranulation with less intensive Toluidine Blue staining and the appearance of ‘ghost’ cells (Fig. 4c), mast cell in the brain of IVIG treated animals were well stained as a sign of granulated, deactivated mast cells (Fig. 4d).

### The prevention of mast cell degranulation resulted in the decreased release of mast cell mediator and less brain inflammation

**Figure 5:**
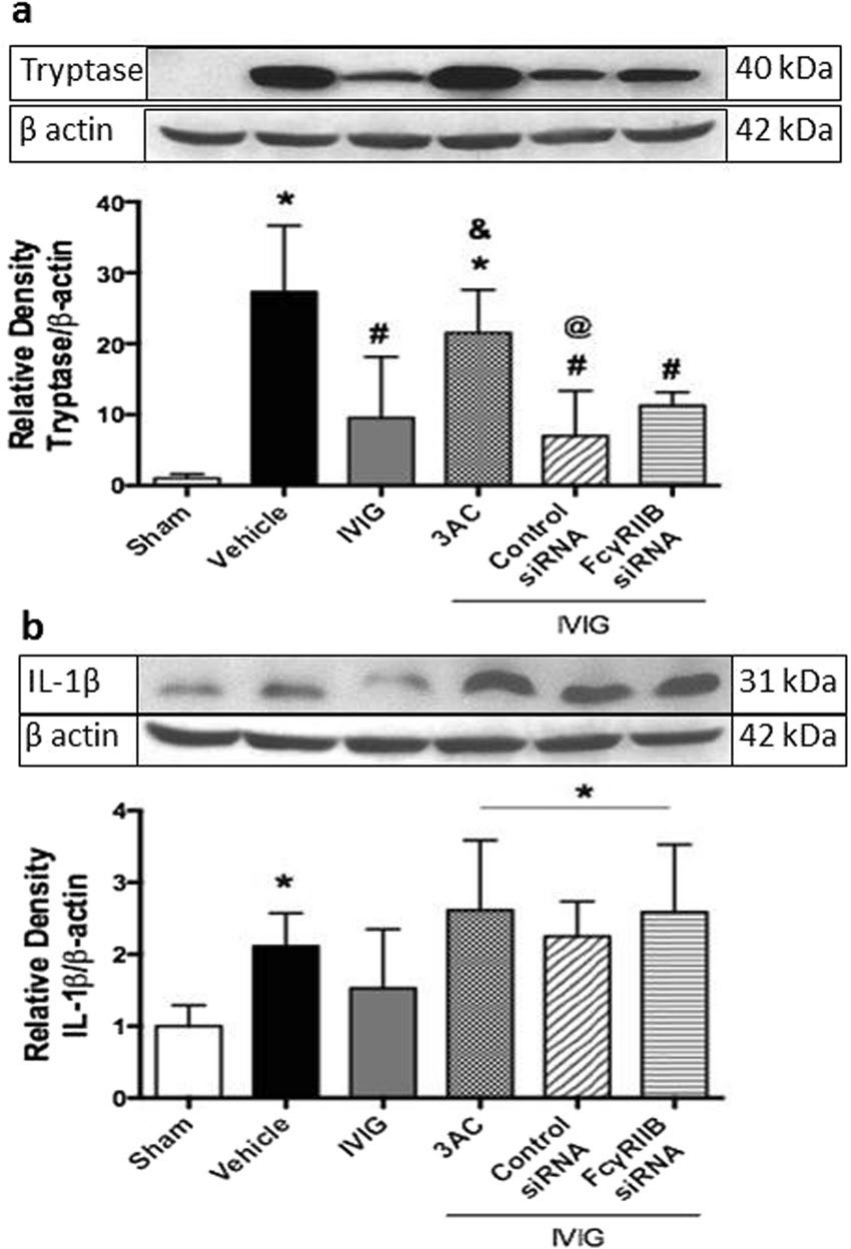
Effects of IVIG treatment on the trypthase release and brain inflammation 24 hours after ICH. ICH caused significant increase of tryptase release (**a**) consequently leading to upregulation of pro inflammatory cytokine release, Il-1β (**C**) IVIG treatment (2 g/kg) attenuated ICH-induced release of tryptase consequently reducing ICH induced inflammatory response. SiRNA to FcγRIIBsiRNA and inhibitor of SHIP1 reverse anti-inflammatory effects of IVIG treatment. SHIP1 inhibtion decreased SHIP expression (**b**) The regions of interests on the membranes were separated and proceeded as described in “Material and Methods” section. Sham n = 6, vehicle n = 6, IVIG n = 6, IVIG + 3AC n = 6, IVIG + control siRNA n = 6, IVIg + FcγRIIBsiRNA n = 6). Values are expressed as mean ± SD. *significant vs. sham, ^#^significant vs. vehicle, ^&^significant vs. IVIG, ^@^significant vs. 3AC, p < 0.05 ANOVA, Tukey Test (**a**,**b**), Student-Newman-Keuls Test (**c**).

Treatment with high dose of IVIG (2 g/kg) decreased ICH-induced release of tryptase (Fig. 5a) 24 hours after ICH. This decrease resulted in the attenuation of ICH-induced brain inflammation, evaluated by western blot to IL-1β (Fig. 5b). Inhibition of the FcγRIIB receptor reversed the beneficial effects of IVIG treatment, increasing production of IL-1β. Furthermore inhibition of FcγRIIB downstream, SHIP1, resulted in the increased production of IL-1β (Fig. 5a,b).

**Figure 6:**
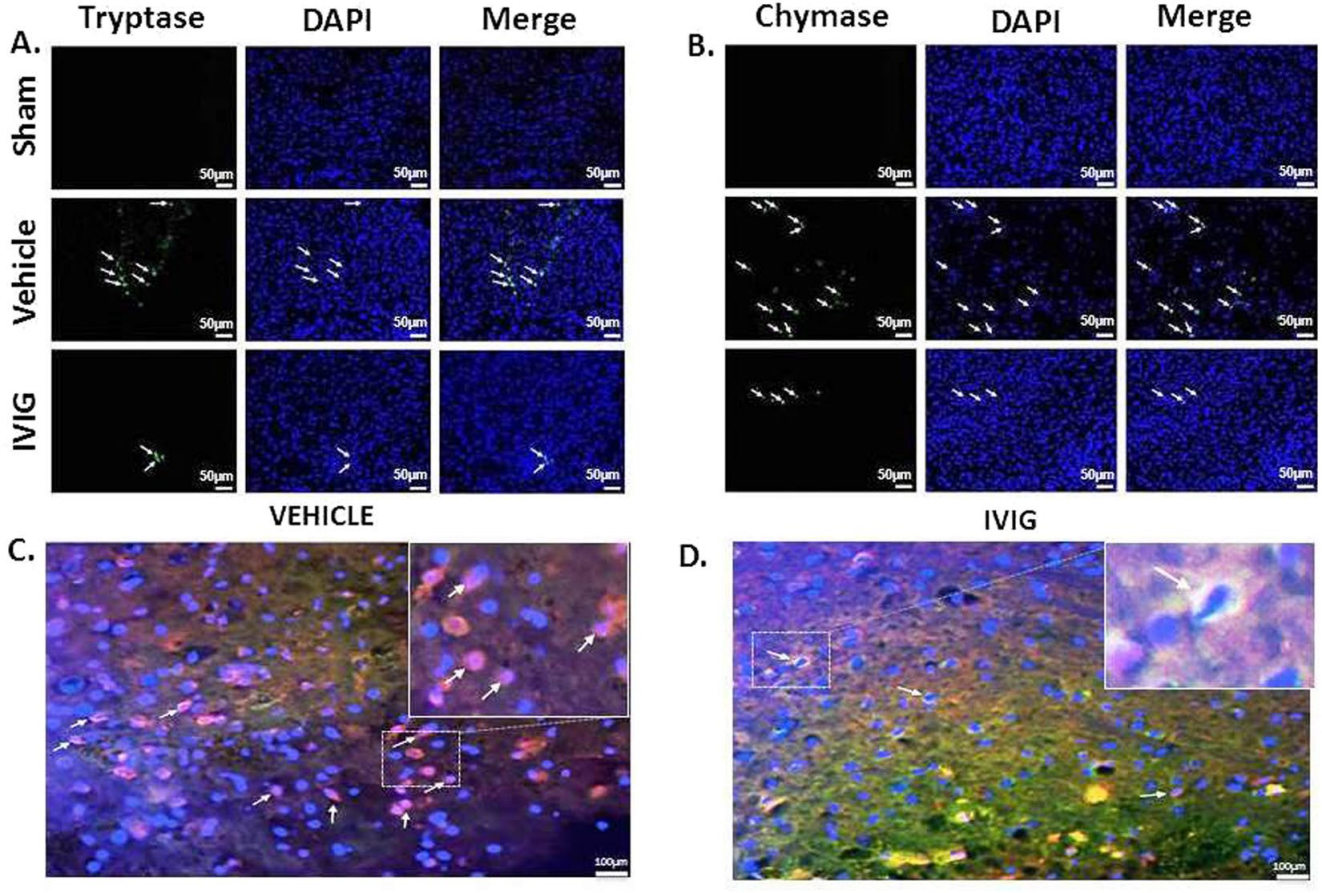
Effect of IVIG treatment on mast cell mediator release. More mast cell mediator positive cells were observed in brain of ICH compared to sham operated animals, IVIG attenuated number of mast cells in brain of ICH animals. Representative immunostaining of (**A**) tryptase and (**B**) chymase positive cells in brain of sham, vehicle, IVIG (2 g/kg) groups at 24 hours. Immunostaining of PI(3,4,5)P_3_ and tryptase in mast cells in vehicle (**C**) and IVIG groups (**D**) at 24 hours. (colocalization for mast cells are shown with white arrows: PI(3,4,5)P_3_: red, tryptase: green, DAPI: blue, 25μm).

Effects of ICH and treatment on mast cell activation were evaluated by immunostaining. While ICH increased the number of tryptase (Fig. 6A) and chymase (Fig. 6B) positive cells, the high concentration of IVIG decreased the effect of ICH.

### IVIG Decreased PI(3,4,5)P3 Expression by Immunostaining

Since IVIG-induced activation of FcγRIIB recruits SHIP, leading to hydrolyzation of PI(3,4,5)P_3_, we tested effect of IVIG treatment on PI(3,4,5)P_3_ in our model and found out that the IVIG treatment decreased the number of PI(3,4,5)P_3_ positive cells (Fig. 6C,D) evaluated 24 hours after ICH.

## Discussion

In the present study we investigated the ability of IVIG to inhibit the ICH-induced mast cell activation and consequently decrease post-ICH inflammation, leading to the preservation of BBB and the improvement of neurological functions after experimental ICH on mice. To the best of our knowledge, that is the first study investigating effects of IVIG on ICH-induced mast cell activation and on the development of the brain injury after ICH.

Mast cells are resident cells in several types of tissues including central nervous tissue. They are located perivascularly, close to neurons and functionally associate with neurons^22,23^. Mast cells include granules containing substances as histamine, heparin, TNF-α and mast cell specific mediators tryptase and chymase^24^. Release of these substances may contribute to ICH-induced inflammatory reactions leading to disruption of BBB, brain edema and neurological dysfunctions. The pharmacological or genetic approaches, leading to mast cell inhibition attenuate development of secondary brain injury after ICH^6,12^.

Mast cells activity is controlled by Fcγ receptor family, which consists of several activating and one inhibitory receptor, FcγRIIB^25^. The FcγRIIB participates in three inhibitory responses. In the most prominent response, the activated receptor recruits Src homology 2-containing inositol 5′ phosphatase (SHIP), in particular SHIP1, which leads to hydrolyzation of PIP3 and release of membrane proteins such as Btk and PLCγ. That results in inhibition of calcium mobilization, needed to mast cells degranulation^26^.

Commercially available IVIG is derived from the blood plasma of healthy individuals and mostly consists of IgG^27^. A major factor underlying an anti-inflammatory property IVIG is IgG-induced activation of the FcγRIIB receptor^20^. The neuroprotective effects of IVIG have been studied in different rodent stroke models. However, effects of IVIG on the development of brain injury after ICH have not been investigated yet.

In the present study we first investigated whether intraperitoneal administration of high molecular weight IVIG would be able to delivery significant amount of the drug into the blood stream of the animals. We observed significant increase of human IgG (active component of IVIG) in the blood of mice. The effect was dose dependent, more human IgG was detected in blood of mice after administration of high dose compared to administration of low dose of IVIG. These results concur with other publications which demonstrated that the IVIG is effective after intraperitoneal administration^28,29^

Furthermore, we examined the dose and time dependent effects of IVIG on ICH-induced injury. We demonstrated that high dose (2 g/kg) of IVIG preserved BBB leading to the decrease of ICH-induced elevation of brain water content 24 hours after ICH. At the same time-point we investigated effects of IVIG on BBB integrity using Evans Blue assay. In agreement with previous publications, ICH resulted in significant accumulation of the Evans Blue stain in ipsilateral hemisphere compared to sham-operated animals^6^. IVIG treatment preserved BBB and resulted in the decreased Evans Blue stain accumulation in brain of treated compared to untreated animals. In cohort with the improvement of BBB integrity, IVIG treatment resulted in attenuation of ICH induced neurological dysfunctions. Beneficial effects of IVIG were also observed in a delayed time point, 72 hours after ICH. At this time point high dose of IVIG significantly decreased BBB disruption, evaluated by Evans Blue assay, as well as improved neurological functions of treated animals. No effects on ICH induced brain edema was observed in this time point. That agreed with previous publication, demonstrated that although mast cell stabilization in acute stage of ICH, it has no effect on brain edema in sub-acute (72 hours after ICH) stage of ICH^6,30^.

The IVIG treatment did not change hematoma volume evaluated by hemoglobin assay at 24 or 72 hours after ICH. It contradicts previous report, stating that mast cell stabilization is able to decrease hematoma volume after ICH^12^. However, it is worth mentioning that the authors of the previous publication used “blood” model of ICH, which employs autologous blood injection into the brain of animals. Despite significant advantages of this model, it is incapable to reproduce re-bleeding and hematoma expansion, which happens in more than 20% of human patients^31^. In order to mimic re-bleeding we have chosen “collagenase” model of ICH for our study. The hallmark of the “collagenase” model is a hematoma expansion and the re-bleeding can be observed up to 24 hours after ICH induction^32^. One can assume that mast cell stabilization may decrease initial volume but not the expansion of the hematoma seeing in “collagenase” model of ICH. To note, authors of the previous publication investigated effect of mast cell stabilization on the hematoma volume by calculation of the volume between at 30 minutes and 24 hours in the same animals. No comparisons of hematoma in treated *vs* untreated animals were done. In our study, we compared hematoma volume in treated vs none treated animals. We were not able to evaluate time dependent expansion of hematoma using the same animal in different time point. Methodical variances of these studies do not allow us to compare our results with the results of previous study directly.

Further we investigated effects of ICH on mast cell activation by western blot study. We observed significant upregulation of mast cell specific mediator, tryptase as early as 3 hours after ICH. Earlier time points have not been investigated. At the same time point, tendency for upregulation was observed by another mast cell mediator, chymase. Earlier accumulation of mast cell mediators is in agreement with previous study postulating that mast cells are first responder for stroke and their activation can be seen in very early (within one hour) stage of disease^33^. Statistic significant upregulation of both tryptase and chymase expression was observed 24 hours after ICH. This time point was used for further investigation.

In order to confirm that protective effects of IVIG are related to it’s ability to stabilize mast cells, we investigated if IVIG treatment will decrease ICH-induced release of mast cell mediators and/or numbers of degranulated mast cells. We demonstrated that the treatment attenuated the ICH-induced tryptase overproduction and reduced numbers of tryptase and chymase positive cells. Furthermore mast cells were visualized by Toluidine Blue staining similarly as others did^11^. While in ICH untreated animals most mast cells were marginally stained and had an appearance of “ghost” cells, the sign of cell activation and degranulation, mast cells in brains of IVIG treated ICH animals were intensively stained and could be identified by their metachromatic cytoplasmatic granules, typical for inactive, granulated mast cells.

Finally we investigated effects of IVIG treatment on PIP3 production. Mast cells were visualized by immunostaining to tryptase. In general tryptase expression was not high. That is in agreement with previous publications, which demonstrated low expression of tryptase in brain^6,34,35^. We hypothesize that the direct damage of BBB is significant but is not only resulted from the action of mast cells. Mast cells are able to activate the most abundant cell type in CNS, microglia^36^. Activated microglia release pro-inflammatory cytokines and produced ROS, triggering development of brain injury after ICH. Hence the IVIG induced stabilization of mast cells may have synergetic effects, attenuating both mast cells and microglia induced brain injury after ICH. In addition, a double staining using tryptase and PIP3 antibody visualized activated mast cells. IVIG treatment decreased number of tryptase/PIP3 positive, activated mast cells.

Although some publications indicated that FcγRIIB activation may negatively affect the mast cell proliferation, we did not observe visible effect of FcγRIIB activation (*via* IVIG) on mast cell proliferation after ICH (Fig. 4)^37^. The c-Kit/SCF pathway is a major pathway involved in mast cell proliferation^38^. The pathway is able to induce mast cell survival by suppressing apoptosis^39^. Furthermore there is an obvious contra play between c-Kit/SCF and immunoglobulin dependent pathway. Pre-incubation of mast cells with SCF significantly increased IgE-induced release of mast cell mediators^40^. Malbec *et al*. demonstrated that FcγRIIB is able to block mast cell proliferation, induced by Kit receptor^37,41^. However we did not observed this effect in our study. We hypothesize that, although the Kit is the major pathway inducing mast cell proliferation, there are other receptors which are also able to activate mast cells^42^. We hypothesize that the blocking of only one pathway, leading to mast cell proliferation was not sufficient to reduce mast cell proliferation after ICH.

Those findings leads us to the conclusion that IVIG treatment inhibited ICH-induced mast cell activation, decreased mast cell degranulation and releasing mast cell mediators, without affecting mast cell proliferation. In order to investigate the molecular mechanisms underlying IVIG induced protection we generated *in-vivo* FcγRIIB receptor knockdown using si-RNA and inhibited major downstream of the receptor, SHIP 1 via small molecule inhibitor 3AC. Knockdown of the FcγRIIB receptor abolished protective effects of IVIG and resulted in increased brain inflammation, brain edema and aggravation of neurological deficits compared with IVIG treated animals. That is in agreement with others, who demonstrated that protective effect of IVIG was mediated by IVIG-induced activation of the FcγRIIB and that knock-down of the receptor abolished IVIG responses^21^.

Anti-inflammatory effects of FcγRIIB receptor stimulation are mediated by activating of SHIP1. In this study we used an inhibitor of SHIP1, AC3^43^. We demonstrated that AC3 decreased expression of SHIP1 in treated animals. Compared to IVIG-treated animals, the inhibition of SHIP1 led to increased release of mast cell mediator and brain inflammation, resulting in increased BBB permeability and aggravation of neurological deficits. That was in agreement with other publications demonstrating that, compared to wild type, SHIP −/− mice exhibit higher levels of mast cell degranulation and inflammatory cytokines production^44^. Moreover our observed is consistent with the observation that administration of a SHIP1 agonist, AQX016 attenuated LPS induced release of TNF-α in mouse model of endotoxemia^45^.

Finally, we investigated effects of IVIG-induced stabilization of mast cells on the production of pro-inflammatory cytokines. Mast cell activation resulted in bi-phasic increase of the pro-inflammatory cytokine production. A rapid release of cytokines upon mast cell degranulation followed by long-lasting increase due to *de novo* syntheses^46^. Furthermore TNFα, released by mast cells stimulated production of IL-1β by another type of cells especially by macrophages^47^. Resident macrophages of the brain, microglia, account for 10–15% of all cells found within the brain. Microglia activation was observed shortly after ICH^48–51^. We investigated effects of IVIG administration on IL-1β production after ICH and demonstrated that IVIG attenuated ICH induced increase of IL-1β production. Pharmacological inhibition of SHIP or *in-vivo* knockdown of FcγRIIB reversed beneficial effects of IVIG. We hypothesize that mast cell stabilization decreased production of IL-1β by mast cells and attenuated IL-1β production by activated microglia. Further investigation of mast cell effects on microglia activation are needed.

In conclusion: IVIG treatments reduced ICH-induced activation of mast cells and consequently decreased brain inflammation, improved integrity of BBB and resulted in the recovery of neurological function after ICH. The protective effects of IVIG treatment were, at least, partly mediated by IVIG-induced activation of the inhibitory receptor of mast cells, FcγRIIB. Since IVIG is a FDA approve drug, the results of the present study are highly translatable into the clinical praxis and represent a promising therapeutic approach, able to decrease post-ICH mortality and improve the life quality of ICH survivals.

## Material and Methods

All procedures were approved by the Institutional Animal Care and Use Committee (IACUC) at Loma Linda University and conducted according to the guidelines for Animal Experimentation at Loma Linda University. A total of 178 CD1 mice (8 week-old male, 30–35 g; Charles River, Wilmington, MA) were used. The animals were housed in a light and temperature controlled environment with unlimited access to food and water.

### Intracerebral hemorrhage induction

ICH was induced via a stereotactically guided injection of collagenase into right basal ganglia as previously described^52^. Briefly, mice were anesthetized with ketamine (100 mg/kg) and xylazine (10 mg/kg, intraperitoneal injection) and positioned prone in a stereotaxic head frame (Stoelting, Wood Dale, IL, USA). An electronic thermostat-controlled warming blanket was used to maintain the core temperature at 37 °C. The calvarium was exposed by a midline scalp incision from the nasion to the superior nuchal line, and the skin was retracted laterally. With a variable speed drill (Fine Scientific Tools, Foster City, CA, USA) a 1.0 mm burr hole was made 0.9 mm posterior to bregma and 1.45 mm to the right of the midline. A 26-G needle on a Hamilton syringe was inserted with stereotaxic guidance 4.0 mm into the right deep cortex/basal ganglia at a rate 1 mm/min. The collagenase (0.075 units in 0.5 µl saline, VII-S; Sigma, St Louis, MO, USA) in the syringe was infused into the brain at a rate of 0.25 µl/min over 2 minutes with an infusion pump (Stoelting, Wood Dale, IL, USA). The needle was left in place for an additional 10 minutes after injection to prevent the possible leakage of collagenase solution. After removal of the needle, the incision was closed and the mice were allowed to recover. Mice were subjected to sham operation received only needle insertion.

### Treatment regimen and interventions

IVIG (Gammagard Liquid-Baxter) was administrated intraperitoneally (i.p), one hour after ICH. Two doses (0.5 and 2.0 g/kg) were tested. The effectivity of intraperitoneal injection for high molecular weight drug (molecular weight of IVIG is ~ 300 kDa) was tested by ELISA (Abcam, ab 195215). Animals were treated with 0.5 or 2,0 g/kg. 24 hours after drug administration animals were anesthetized and blood were collected using inferior vena cava. The level of human IgG (active component of IVIG) in mice was investigated according to vendor’s recommendation. 6 animals per group and 6 naïve animals were used

SHIP1 inhibitor, 3α-aminocholestane (3AC), (Echelon Biosciences, 30 mg/kg) was administrated 30 minutes post-ICH, intraperitoneally. siRNA FcγRIIB or control (scrambled) RNA (OriGene Technologies, 100 pmol/2 μL) was given via intracerebroventricular (i.c.v) injection 24 hours before ICH.

### Experimental groups

In the first experiment, animals were divided into four groups a) sham (a needle trauma only), b) ICH animals treated with saline (vehicle), c) ICH animals treated with 0.5 g/kg IVIG, one hour after ICH, d) ICH animals treated with 2 g/kg IVIG, one hour after ICH. 23 and 71 hours after ICH, animals were tested neurologically. One hour after the neurological testing, animals were sacrificed and brain water content was measured. Additionally at these time points, 6 sham animals, 25 ICH animals treated with vehicle or with IVIG (2 g/kg) were used for evaluation of the effect of IVIG on the hematoma volume (hemoglobin assay) and BBB integrity (Evans Blue assay).

In the second experiment, animals were divided into 6 groups: sham, 3, 6, 12, 24, and 72 hours after ICH for measuring the expression of tryptase and chymase.

In the third experiment, animals were divided into 6 groups a) sham, b) ICH animals treated with vehicles, c) ICH animals treated with 2 g/kg IVIG, d) ICH animals treated with IVIG and SHIP1 inhibitor 3AC (30 mg/kg i.p 30 min post ICH), e) ICH animals treated with IVIG and control siRNA (100 pmol/2 μl, i.c.v 24 h before ICH), f) ICH animals treated with IVIG + FcγRIIB siRNA (100 pmol/2 μl, i.c.v). Twenty-three hours after ICH, animals were tested for neurological function, brain water content evaluation. Additionally tryptase, SHIP1, IL-1β expressions were evaluated by western blotting.

In the fourth experiment, animals were divided into 3 groups, a) sham, b) ICH animals treated with vehicle, c) ICH animals treated with 2 g/kg IVIG. Animals were sacrificed at 24 hours after ICH induction. Brain samples were used for evaluation of IVIG effect on mast cell degranulation, tryptase/chymase/PIP3 expression after ICH.

### Assessment of neurological deficits

Neurological functions were assessed by an independent researcher blinded to the procedure 23 and 71 hours after ICH. Four tests were implemented for evaluation of neurological functions, including modified Garcia test, wire hanging, beam balance and limb placement, as previously described^52–55^.

### Evaluation of BBB integrity and hematoma volume

Mice were euthanized 24 or 72 hours post ICH. BBB integrity was measured by brain water content as previously described^56^. Briefly, mice were decapitated under deep anesthesia. The brains were immediately removed and dissected into four parts: ipsilateral and contralateral basal ganglia and cortex. The cerebellum was collected as an internal control. Each part was weighed on an electronic analytical balance (APX-60, Denver Instrument, New York, NY, USA) giving the *WW* (wet weight) and then dried at 100 °C for 24 hours to determine the *DW* (dry weight). The brain water content (%) was calculated as [*(WW – DW*)/*WW*] × 100.

Evans Blue assay was also used for evaluation of BBB permeability as previously described^57^. Briefly, a 2% solution of Evans Blue in normal saline (4 ml/kg of body weight) was injected intraperitonally. Three hours after the injection, the mice were transcardially perfused with ice-cold PBS (pH 7.4) and the brains were collected. Evans Blue stain was measured by spectrophotometer (Thermo Spectronic Genesys 10 UV, Thermo Fischer Scientific Inc., Waltham, MA, USA) at 610 nm. The results are presented as (µg of Evans Blue stain)/(g of brain).

The hematoma volume was quantified by hemoglobin assay as previously described^56,58^. Hemispheric brain tissue was obtained from mice subjected to complete transcardial perfusion to remove intravascular blood. Brain tissue was homogenized in PBS for 30 seconds followed by sonication for 1 minute and centrifugation at 15,000 rpm for 30 min (4 °C). Drabkin’s reagent (0.4 ml, Sigma) was added to 0.1 ml supernatant aliquots and allowed to stand for 15 min at room temperature. Optical density was measured and recorded at 540 nm with a spectrophotometer.

### Western blot analysis

Mice were perfused transcardially with 40 ml of cold PBS. Hemispheres were isolated and stored at −80 °C until analysis. Protein extraction and western blots were performed as previously described^52^. Briefly, the whole-cell lysates were obtained by homogenizing in RIPA lysis buffer (Santa Cruz Biotechnology, Inc., sc-24948) and centrifuging (14,000 g at 4 °C for 30 min). Equal amounts of protein (50 mg) were loaded and subjected to electrophoresis on an SDS-PAGE gel. After being electrophoresed and transferred to a nitrocellulose membrane. To separate the region where the target protein will appear, the membrane was cut along the molecular weight marker. The member straps were then blocked and incubated with the primary antibody overnight at 4 °C. Following primary antibodies were used: anti-tryptase 1:1000 (Santa Cruz Biotechnology, sc-32889), anti-chymase 1:100 (Abcam, ab186417), anti- IL-1β (Abcam, ab9722) 1:750 and anti SHIP1 1:500 (Santa Cruz Biotechnology, sc-6244). The antibody against β-actin (Santa Cruz, 1:1000) was used as the internal control.

If the target protein had similar (+/− 20 kDa) weight compared to β-actin, the membrane straps were blocked and proceeded as described above.

Some representative strips were proceeded with “Microsoft Office 2010”

### Immunofluorescence staining

At 24 hours after ICH, mice were perfused under deep anesthesia with cold PBS, followed by infusion of 4% paraformaldehyde^59^. The brains were then removed and fixed in formalin at 4 °C overnight followed by dehydration with 30% sucrose in PBS. The frozen coronal slices (10 mm thick) were sectioned in cryostat (CM3050S; Leica Microsystems, Bannockburn, IL, USA). Brain slice were hydrated (30 minutes bi-distillated water room temperature) and stain with fresh prepared Toluidine Blue solution (0.1%, pH = 2.0) for three minutes. The slices were washed with distillated water three time, dehydrated through 75%, 95% and 2 changes of 100% alcohol, cleared in xylene substitute and coverslip with mounting medium. The perihematomal region of coronal brain sections were incubated overnight at 4 °C with the following primary antibodies: anti-tryptase 1:100 (Santa Cruz Biotechnology, sc-32889), anti-chymase 1:100 (Abcam, ab186417), anti-PIP3 1:100 (Abcam, ab11039), followed by incubation with appropriate FITC- conjugated secondary antibodies (Jackson ImmunoResearch). Sections were observed under an OLYMPUS BX51 microscope with fluorescence light.

### Statistical Analysis

Data are expressed as mean ± SD. All data were analyzed by one-way ANOVA, followed by the Tukey test or Student-Newman Keuls. A p value of < 0.05 was considered as statistically significant.

## Electronic supplementary material

Supplemental materials
