# Supplementary material for: IVIG activates FcγRIIB-SHIP1-PIP3 Pathway to stabilize mast cells and suppress inflammation after ICH in mice
Source: Sci Rep. 2017 Nov 14;7:15583. doi: 10.1038/s41598-017-15455-w (PMC5686215; doi:10.1038/s41598-017-15455-w)
Supplement: Supplementary file 1 — Supplemental materials [file 41598_2017_15455_MOESM1_ESM.pdf]

IVIG activates FcγRIIB-SHIP1-PIP3 Pathway to stabilize mast cells and suppress inflammation after ICH in mice

Gokce Yilmaz Akyol[1*], Anatol Manaenko[1,2*], Onat Akyol[1], Ihsan Solaroglu[1], Wing Mann Ho[1], Yan Ding[1], Jerry Flores[1], John H. Zhang[1,3], Jiping Tang[1*]

[1]Departments of Basic Science, Loma Linda University, Loma Linda, CA, USA; [2]Departments of Neurology, University of Erlangen-Nuremberg, Erlangen, Germany; [3]Department of Anesthesiology, Loma Linda University, Loma Linda, CA, USA

[*]Correspondence to Jiping Tang, MD, Department of Basic Science, Loma Linda University School of Medicine, 11041 Campus St, Loma Linda, CA 92354. E-mail: jtang@llu.edu

[*] These authors contributed equally to this work

Supplementary material

The proposed pathway leading to IVIG-induced stabilization of mast cells:

A well-established way of mast cell activation includes phosphorylation of PIP2 resulting in the increased production of a secondary messenger, PIP3, and consequently leading to PLC γ activation (Barker et al., 1999; Oppong et al., 2013). The activated PLC γ degradates PIP2 resulting in accumulation of IP3 and subsequently in the increase of intracellular Ca^{2+} concentration. The increase Ca^{2+} concentration induces mast cell activation, leading to the release of mast cell mediators (Hayama et al., 2011; Sun et al., 2016).

IVIG activates FcγRIIB, an inhibitory receptor. The activation of FcγRIIB results in the activation of SHIP1, leading to the attenuation of PIP3 production and thus to the decreased IP3 accumulation (Tanigaki et al., 2009). These decrease the intracerebral Ca^{2+} concentration, stabilizing mast cells and decreasing release of mast cell mediators.

In our project we hypothesized that ICH-induced activation of mast cells is mediated, at least partly, by the increase of PIP3 production. IVIG, via SHIP1 activation, decreases the PIP3 production, resulting in the mast cell stabilization and in the decrease of mast cell release. These results in less brain inflammation, improved functions of BBB and consequently in a smaller post-ICH brain injury and better neurological functions.

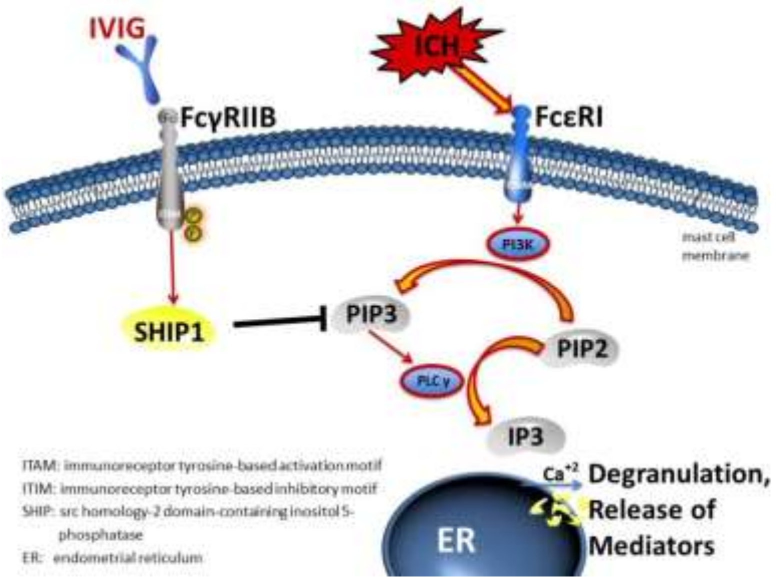

Supplemental Figure 1

IVIG activates inhibitory receptor FcγRIIB, leading to SHIP activation, attenuation of PIP production and consequently to mast cell stabilization

Activation

Phosphorylation / Dephosphorylation

Blockade

Effect of intraperitoneal IVIG administration on the level of human IgG in blood of mice

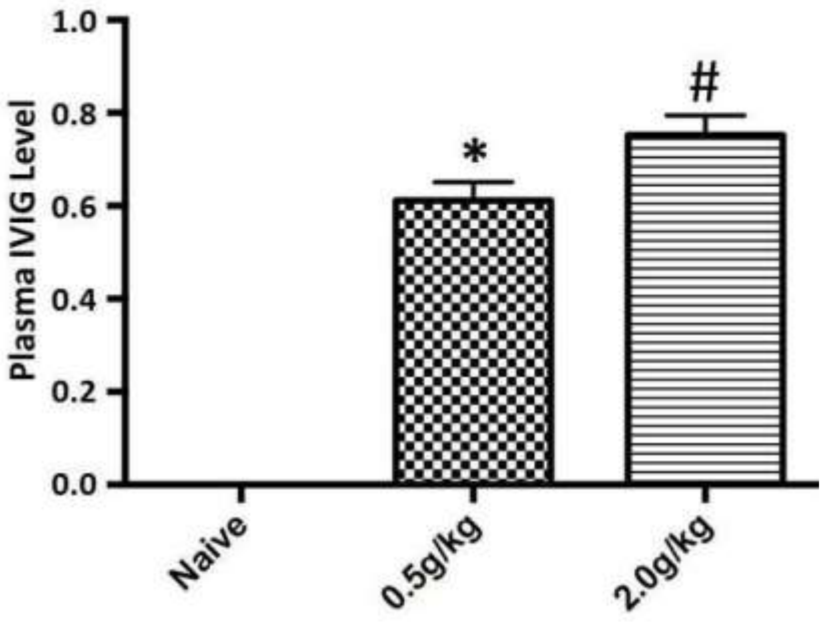

Supplemental Figure 2

i.p. administration of IVIG resulted in dose-dependent increase of human IgG in blood of mice 24 hours after administration. 6 mice per group were used. Values are expressed as mean ±SEM.

* significant vs. sham,

# significant vs. sham and 0.5 g/kg dose, $p<0.05$ ANOVA, Tukey Test.

IVIG treatment has no effects on hematoma volume

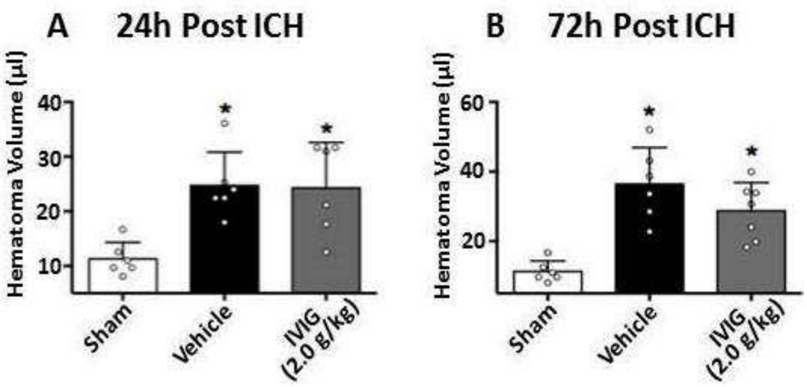

Supplemental Figure 3

IVIG does not affect hematoma volume evaluated at 24 (panel A) or at 72 (panel B) hours after ICH (all groups N=6, IVIG 72 hours N=7). Values are expressed as mean ±SEM, * significant vs. sham, $p<0.05$ ANOVA, Tukey Test.

Table 1: Effects of ICH and pharmacologic manipulations on the mortality rate of animals

Groups	Total Number	Number of deads	Mortality rate in %
Sham	20	0	0
Vehicle	74	10	13.5
Low dose i.p.	6	0	0
High dose i.p.	39	5	12.8
3AC i.p.	12	0	0
Control siRNA	14	2	14.3
fCgsiRNA	14	2	14.3
TOTAL	179	19	10.6

Naïve animals used for examination of IVIG level in blood of mice (Supplemental Figure 2) are **not** included in the table

Barker, S. A., et al., 1999. Multiple roles for PI 3-kinase in the regulation of PLCgamma activity and Ca2+ mobilization in antigen-stimulated mast cells. J Leukoc Biol. 65, 321-9.

Hayama, K., et al., 2011. Gold activates mast cells via calcium influx through multiple H2O2-sensitive pathways including L-type calcium channels. Free Radic Biol Med. 50, 1417-28.

Oppong, E., et al., 2013. Molecular mechanisms of glucocorticoid action in mast cells. Mol Cell Endocrinol. 380, 119-26.

Sun, R., et al., 2016. Calcium Influx of Mast Cells Is Inhibited by Aptamers Targeting the First Extracellular Domain of Orai1. PLoS One. 11**,** e0158223.
Tanigaki, K., et al., 2009. C-reactive protein inhibits insulin activation of endothelial nitric oxide synthase via the immunoreceptor tyrosine-based inhibition motif of FcgammaRIIB and SHIP-1. Circ Res. 104**,** 1275-82.
